# Use of verbal autopsy in a national health information system: Effects of the investigation of ill-defined causes of death on proportional mortality due to injury in small municipalities in Brazil

**DOI:** 10.1186/1478-7954-9-39

**Published:** 2011-08-04

**Authors:** Elisabeth França, Deise Campos, Mark DC Guimarães, Maria de Fátima M Souza

**Affiliations:** 1 Graduate Program in Public Health and Research Group in Epidemiology and Health Evaluation, Faculty of Medicine, Federal University of Minas Gerais. Av. Alfredo Balena, 190. Belo Horizonte, 30130-100, Brazil; 2 Research Group in Epidemiology and Health Evaluation, Faculty of Medicine, Federal University of Minas Gerais, Brazil. Av. Alfredo Balena, 190. Belo Horizonte, 30130-100, Brazil; 3 Department of Social Medicine and Graduate Program in Public Health, Faculty of Medicine, Federal University of Minas Gerais; Research Group in Epidemiology and Health Evaluation, Faculty of Medicine, Federal University of Minas Gerais, Av. Alfredo Balena, 190. Belo Horizonte, 30130-100, Brazil; 4 Area of Health Surveillance and Disease Prevention and Control, Pan American Health Organization. 525 Twenty-third St. N.W., 20037-2895, Washington, DC

## Abstract

**Background:**

The Mortality Information System (MIS) in Brazil records mortality data in hospitals and civil registries with the responsibility of compiling underlying cause of death. Despite continuous improvements in the MIS, some areas still maintain a high proportion of deaths assigned to ill-defined causes. Deaths coded to this category have most likely been considered as miscoded deaths from communicable and noncommunicable diseases. However, some local studies have provided evidence of underreporting of injury in Brazil. The aim of this study was to investigate ill-defined causes of death using the verbal autopsy (VA) method to estimate injury-specific mortality fraction in small municipalities in northeastern Minas Gerais, Brazil.

**Methods:**

A sample size of reported death certificates with ill-defined conditions in a random sample of 10 municipalities was obtained, and then trained interviewers questioned family members using a standardized VA questionnaire to elicit information on symptoms experienced by the deceased before death. All attempts were made to collect existing information about the disease or death using health facilities records. Probable causes of death were assigned by a physician after review of the completed questionnaires following rules of the 10^th ^revision of the International Classification of Diseases (ICD-10).

**Results:**

Of 202 eligible ill-defined deaths, 151 were investigated using the VA methodology, and 12.6% had injury as the underlying cause of death. The proportional mortality fraction from injury among all causes of death increases from 4.4% to 8.2% after investigation. Different specific injury category causes were observed between recorded injury causes and those detected by VA. Drowning was the top specific injury cause detected after investigation.

**Conclusions:**

This study provides evidence that the use of VA in the investigation of registered ill-defined conditions in an existing MIS can furnish information on the relevance of injury as a priority health problem in small municipalities of Minas Gerais. Local research with VA should be brought to the attention of regional health policymakers to improve the quality of data for their planning.

## Introduction

Mortality data obtained from vital statistics registration systems (VR) are critical for rational public health planning. However, their usefulness will depend on the quality of death certificates, with emphasis on the reliability of cause-specific mortality data. Indicators of the quality of VR include completeness of registration and the proportion of deaths coded as ill-defined condition [1].

In Brazil, the Mortality Information System (MIS) is one of the key components of the National VR, and it was entrusted with the active recording of mortality data in hospitals and registry offices. The MIS is responsible for compiling cause of death data throughout the country, with a current estimate of more than 1 million coded deaths annually. Despite the continuous improvements of the MIS, Brazil is classified as having medium-quality death registration data, based on estimates of the national level of registration completeness and the proportion of ill-defined causes of death [1].

Despite the high proportion of ill-defined conditions in Brazil, up to 2010 there were only two published studies that investigated the distribution of defined causes of death among ill-defined conditions [2,3]. In both studies, 4-5% of these conditions were found to be due to injury, which indicates an unusual result, as more complete registration from injuries would have been expected [2,4].

During the first decade of this century, the Ministry of Health implemented a series of policies focused on helping states of the north and northeast regions to reduce ill-defined causes of death. Since 2005, there has been a substantial decline in the proportion of ill-defined causes in these regions, particularly in the northeast. In 2007, the level of completeness was estimated to be 90% for the whole country, and 7.7% of deaths were assigned to symptoms, signs, and ill-defined causes of death [5,6]. In spite of the steady decrease in the proportions of ill-defined conditions, these still remain high in several specific areas of the country [7].

Although some initiatives for investigating ill-defined causes of death reported to MIS by health professionals used hospital records, health department records, and interviews with relatives of the deceased [5,8], until 2008 there was no national standard procedure for this investigation. In 2007, the Ministry of Health began to discuss the adoption of adapted verbal autopsy (VA) questionnaires developed by the World Health Organization (WHO) for the investigation of ill-defined causes of death. As part of these initiatives, we conducted a study to assess the use of VA in the investigation of the causes of underregistered deaths and reported deaths to MIS from symptoms, signs, and ill-defined conditions in the northeast region of Minas Gerais state, Brazilian southeast region. We found that 14% of recorded ill-defined conditions and nonrecorded deaths in 2007 were due to injuries [7].

The finding of injury among ill-defined conditions is a striking issue, as injuries cause a significant health burden in Brazil, even taking undercount into consideration, and the death rates from violence rank among the highest in the Americas [9]. Therefore, proportional mortality for ill-defined causes of death must be considered in the use of data for epidemiological analysis such as burden of disease estimation, with suitable adjustments to account for potential bias. Miscoding of injury to ill-defined causes is of concern, and it may be indicative of an urgent need to check the quality of the VR system. It should also be investigated whether it is necessary to consider injury in the redistribution rules to estimate levels of cause-specific mortality. Thus, the aim of the present study was to investigate in a more detailed way causes of death reclassified as due to injury among those initially recorded as ill-defined causes in the routine information system in the northeast region of Minas Gerais.

## Methods

This is a cross-sectional study of death certificates with ill-defined causes officially reported to the MIS in the northeast region of Minas Gerais state, Brazil. Minas Gerais is located in the southeast region of Brazil with an estimated population of 19 million inhabitants in 2007, and it is divided into 13 health administrative regions. These regions differ widely in living standards and population features. The northeast region has approximately 880,000 inhabitants, and it has the highest burden of disease and the poorest quality of cause-specific mortality data in the state [10].

The sample size was estimated in 164 death certificates (DC) with ill-defined causes to be investigated considering the following parameters: 1) number of total death certificates with ill-defined causes in 2007 in the region reported up to April 2008: 1,124; 2) percentage of anticipated frequency of ill-defined causes to be reclassified into well-defined causes: 80%; 3) absolute precision: 6%; 4) confidence level: 90%; and, 5) design effect: 1.5. We then added 20% due to potential losses, yielding 197 DCs with ill-defined causes for reclassification, which were rounded to 200.

The resident population of this region is geographically dispersed into 63 municipalities as follows: a) 49.2% with fewer than 10,000 inhabitants; b) 31.8% between 10,000 and 19,999 inhabitants; and, c) 19.0% with 20,000 or more inhabitants. In order to obtain the 200 DCs, we used a two-stage sampling procedure. In the first stage, we randomly chose 10 municipalities among the 63, proportional to their population size as indicated above. Assuming that ill-defined causes were distributed similarly among the municipalities, these 10 municipalities would suffice to reach the required sample size. In the second stage, all DCs with ill-defined causes reported in these municipalities were then selected.

Verbal autopsy interviews were conducted with family members of the deceased within 13 months following the date of death, on average. In the majority of cases respondents were family members, such as son/daughter (36% of the total), husband/wife (19%), parents (7%), or other family members (30%). Informed consent was obtained before the interviews, which were carried out by trained interviewers with at least secondary schooling. All interviewers have been carefully trained in interview techniques. Almost all interviews were conducted with the assistance of a community health worker from the Family Health Program who accompanied the interviewer during the home visits. This facilitated the address location and provided emotional support to the family. However, the health worker was not present at the time of the interview because of the possibility of introducing respondent bias and for ethical reasons. A standardized VA questionnaire was used to elicit information on symptoms experienced by the deceased before death. This questionnaire was based on the Portuguese version of the WHO instrument previously used in Mozambique and adapted by the Ministry of Health for the Brazilian reality, including cultural differences and the most prevalent diseases [5,11]. In addition, all attempts were made to trace existing information about the disease or death, including data obtained from hospital, health department, autopsy, family health program, or civil registry office records and by interviewing family health program professionals. Two forms were applied according to the age at death: (i) from 28 days old to younger than 10 years old and (ii) 10 years old or over. As we did not find registered ill-defined causes for neonatal deaths, it was not necessary to use the specific form for this age group. A medical professional trained in cause of death certification assigned the underlying cause of death of each individual using both the VA and the medical records, or any other documented evidence available that might have helped in determining the probable cause of death. Rules of the 10th revision of the International Classification of Diseases (ICD-10) were applied. Causes of death were then classified using a standardized mortality tabulation list from WHO comprised of groupings of ICD-10 codes, including 18 specific items for external causes of death [11].

Descriptive analysis was performed for data from the VA interviews of ill-defined conditions. For diagnosing deaths due to accidental or violent injuries, the criterion used was the report of an accident (intentional or unintentional) that was considered the underlying cause of the death by the physician if it was a logical sequence between the injury and the other causes. We used a classification approach with a few broad categories of injuries to increase the validity of VA. All the data collectors and the medical professionals were blinded to the objective of the study. Persons with injury cases detected among investigated ill-defined conditions were compared to people with injury cases registered on VR using Fisher's exact test to examine whether the range of injury causes and other selected characteristics of death were significantly different between these two groups. Significance level considered was 0.05. We obtained the registered mortality statistics in the VR system from the Brazilian Ministry of Health [12]. Deaths coded to ill-defined conditions in the studied municipalities of Minas Gerais northeast region were reassigned to injury and medical causes according to observed proportions detected among ill-defined causes in the investigated sample. The resultant estimates from these adjustments and the registered VR causes were summed to obtain cause of death estimates. Proportions of deaths from ill-defined causes and injuries, deaths occurring at home in VR data, and demographic information (number of municipalities and proportion of resident population) in selected administrative areas of the country were calculated by municipality size using data made available by the Brazilian Ministry of Health [12]. Data entry and analysis were carried out by Excel and Stata 9.0 statistical package.

Ethical approval for this study was obtained from the Federal University of Minas Gerais Ethical Committee. The study was also implemented with the full knowledge and support of the Brazilian Ministry of Health and the State Health Department of Minas Gerais.

## Results

A sample of 202 (35.3%) ill-defined conditions among 572 deaths was obtained from the 10 municipalities randomly selected. Six cases with defined causes of death at hospital investigation were excluded, and 45 cases could not be located due to migration of households or missing address. Thus, we investigated 151 (74.8%) ill-defined deaths with the verbal autopsy methodology. No statistical difference was found between the participants in the study sample (n = 151) and losses (n = 45) with regard to the proportion of deaths occurring in hospitals and the age distribution of the deceased. On the other hand, being male (76%), living in a rural area (62%), and having no coverage from the Family Health Program (58%) were significantly more frequent in losses.

Table 1 shows the distribution of selected characteristics in the VA interviews of ill-defined conditions. Among the investigated deaths, 12.6% were due to injuries. The majority of people were over 60 years old, the sex distribution was similar, and 81% of the deceased lived in urban areas. Although the majority of deaths occurred at home, 62.9% of the respondents to VA questionnaires informed us that the deceased had visited a health facility during the period in which the terminal events leading to death occurred. This proportion, however, was lower (32.5%) when we checked the health facility registries (data not presented). On the other hand, we should note that 91% of the deceased with a registered ill-defined cause of death were covered by the Family Health Program and also that some deaths took place in a health facility.

**Table 1:** Proportion of injuries and other variables in the sampled municipalities after investigation of ill-defined conditions using verbal autopsy (VA), Minas Gerais northeast, Brazil, 2007

Variables	n (%)
	
Cause of death	
Injury	19 (12.6)
Medical causes	110 (72.8)
Ill-defined	22 (14.6)
	
Age (years)	
0-19	5 (3.3)
20-59	40 (26.5)
≥60	106 (70.2)
	
Sex	
Male	78 (51.7)
Female	73 (48.3)
	
Rural area	
Yes	29 (19.2)
No	122 (80.8)
	
Death place*	
Home	119 (79.3)
Hospital	15 (10.0)
Others	16 (10.7)
	
Having FHP**	
Yes	138 (91.4)
No	13 (8.6)

*missing, not included (n = 1); ** Family Health Program

As Table 2 indicates, different distribution of specific causes within the injury category, sex, and the death place were observed between injury causes coded by the VR system before investigation and those reclassified as injury among the ill-defined conditions by the VA method. Road traffic accidents were the top cause of injury deaths in VR while drowning ranks first in the reclassified injury deaths (VA data). Although the age distribution was relatively similar, female deaths were relatively more common in the VA data if compared with VR data. Thirty-seven percent of injuries detected among ill-defined conditions occurred at home, as compared to none in the VR data. The proportion of residents in rural areas was similar in both groups.

**Table 2:** Injury in vital registration (VR) data before investigation and among investigated ill-defined conditions by the VA method in the sampled municipalities, Minas Gerais northeast, Brazil, 2007

Variables	Injury in VR data before investigation	Injury among ill-defined conditions	p value
	n (%)	n (%)	(Fisher's exact test)
Cause of death			
Road traffic accidents	10 (40.0)	1 (5.3)	0.00
Falls	1 (4.0)	4 (21.1)	
Drowning	2 (8.0)	6 (31.6)	
Exposures* and poisonings	3 (12.0)	1 (5.3)	
Assault	9 (36.0)	4 (21.1)	
Events of undetermined intent	0	3 (15.8)	
			
Age (years)			
1-19	4 (16.0)	2 (10.5)	0.32
20-59	18 (72.0)	11 (57.9)	
60 +	3 (12.0)	6 (31.6)	
Sex			
Male	22 (88.0)	12 (63.2)	0.07
Female	3 (12.0)	7 (36.8)	
			
Death place**			
Home	0	7 (36.8)	0.00
Hospital	6 (25.0)	3 (15.8)	
Others	18 (75.0)	9 (47.4)	
			
Rural***			
Yes	7 (31.8)	5 (26.3)	0.74
No	15 (68.2)	14 (73.7)	

*exposure to fires, venomous animals, force of nature; ** missing, not included (n = 1); *** missing, not included (n = 3)

The proportion of injuries on VR data in the sampled municipalities was 4.4% (n = 25), considering all causes of death (n = 572). Two hundred and two cases were registered as ill-defined causes of death, but six cases had medical records with defined causes and three deaths among them were due to injury. Based on the fact that the number of investigated ill-defined cases is 151 and 19 among them were due to injury, we should have 47 injury deaths. So the injury fraction after investigation should be 8.2%; that is to say, the proportional mortality fraction due to injury among defined causes in the 10 municipalities increased from 4.4% to 8.2% after investigation (Table 3).

**Table 3:** Proportional mortality due to injuries in the sampled municipalities in vital registration (VR) data before and after investigation, Minas Gerais northeast, Brazil, 2007

Cause of death	VR before investigation		VR after investigation	
	n	%	n	%
				
Injuries	25	4.4	47	8.2
				
Medical causes	345	60.3	458	80.1
				
Ill-defined causes	202	35.3	67	11.7
				
Total	572	100.0	572	100.0

Table 4 provides a summary of the proportional distribution of ill-defined causes of death, injuries, and home deaths, and also some demographic characteristics of Brazil and some administrative areas according to municipality size. Although the proportions of injuries in the state of Minas Gerais (11.1%), Brazilian southeast region (11.0%) and Brazil (12.5%) were relatively similar, this proportion in the northeast region of Minas Gerais is 9.2%. Within each area, the fraction of injuries is likely to be directly related to the size of the municipality. We also should point out that while smaller municipalities have a higher proportion of ill-defined conditions and home deaths than larger ones, this pattern is similar throughout the country in all administrative areas, and it is far greater in the northeast region of Minas Gerais.

**Table 4:** Proportion of ill-defined conditions, injuries, home deaths, municipalities, and population by municipality size in selected administrative areas of Brazil, 2007

Administrative area and municipality size (inhabitants)	% Ill-defined	% Injury	% Home deaths	% Municipalities	% Population
					
Minas Gerais northeast					
	(n = 5,409)	(n = 5,409)	(n = 5,409)	(n = 63)	(n = 881,340)
< 50,000	29.8	8.7	35.9	98.4	85.6
50,000-99,999	0.0	0.0	0.0	0.0	0.0
100,000 +	9.5	10.6	22.3	1.6	14.4
Missing*	2.5	2.5	2.5	0.0	0.0
Total	25.8	9.2	32.5	100.0	100.0
					
Minas Gerais state					
	(n = 111,366)	(n = 111,366)	(n = 111,366)	(n = 853)	(n = 19,719,285)
< 50,000	14.3	9.2	25.7	92.1	42.3
50,000-99,999	12.2	10.0	19.2	4.5	13.6
100,000 +	7.5	13.5	16.3	3.4	44.1
Missing*	0.2	0.2	0.2	0.0	0.0
Total	11.2	11.1	20.9	100.0	100.0
					
Southeast					
	(n = 495,877)	(n = 495,877)	(n = 495,877)	(n = 1,668)	(n = 80,641,101)
< 50,000	12.2	9.2	22.0	85.7	22.0
50,000-99,999	11.1	10.3	16.9	6.2	9.3
100,000 +	6.1	11.4	13.8	8.1	68.7
Missing*	0.5	0.5	0.5	0.0	0.0
Total	8.0	11.0	15.9	100.0	100.0
					
Brazil					
	(n = 1,047,824)	(n = 1,047,824)	(n = 1,047,824)	(n = 5,564)	(n = 189,335,191)
< 50,000	10.6	11.3	30.2	89.5	33.8
50,000-99,999	9.0	12.5	23.0	5.6	11.4
100,000 +	5.5	13.0	16.0	4.9	54.8
Missing*	0.4	0.4	0.4	0.0	0.0
Total	7.7	12.5	21.4	100.0	100.0

Source: Ministry of Health (http://www2.datasus.gov.br/DATASUS/index.php; accessed May 2011)

* Record linkage of the population size data bank and the Mortality Information System was not possible.

## Discussion

Our findings indicate that the use of routine vital statistics may underrepresent the burden of external causes in northeastern Minas Gerais, as 12.6% of deaths registered with no defined causes in small municipalities were in fact due to injuries after VA investigation. We also found different proportional composition of injury types in the investigated study sample pointing out that some of them are more likely to be misclassified, e.g., drowning and falls.

Although there have been some previous initiatives in using VA in Brazil, they were focused on the investigation of infant deaths [13]. To our knowledge, this study is one of the first attempts to use VA standardized procedures in the investigation of recorded ill-defined conditions for deaths occurring predominantly outside of hospitals as an adjunct to medical certification in the routine mortality information system in Brazil.

VA is a method of ascertaining probable causes of a death based on an interview with family relatives or caregivers, using a questionnaire to elicit information on signs and symptoms experienced by the deceased before death and also the circumstances preceding that death. It has been used extensively for estimating the causes of death structure of a defined population, mainly in countries with Demographic Surveillance Sites or sentinel/sample vital registration systems, and more recently for verifying registered causes of death in a national VR system [14-17]. In Brazil, it has been proposed as a complement to the VR system for all states with high proportions of home deaths [5]. But it is important to note that VA is a *limited substitute for proper medical certification *[18], and its use in some areas of the country is part of the Brazilian government initiatives to strengthen the VR system [5].

The proportion of ill-defined causes of death in VR in Brazil has been the focus of several studies concerning its characteristics, distribution, and determinants, showing that a higher proportion of ill-defined conditions occur among children (1-4 years old) and the elderly. The majority of cases occurred at home [19-21], and unattended deaths make up the highest proportion of mortality [21-23], except in capitals [24]. In our study, we found that only 10% of the ill-defined deaths occurred in hospitals. Thus, it is likely that hospital investigation would have a smaller impact in decreasing the proportion of ill-defined diseases as suggested from a study in São Paulo [22] and that household investigation using the VA method would be the best option in this case.

As expected, in-hospital deaths were negatively associated with ill-defined conditions [24], even after controlling for socio-demographic characteristics and chronic diseases [25]. In our study when the municipality was adopted as a unit of analysis in an ecological approach, we found that the proportions of ill-defined conditions and home deaths were inversely related to the size of the municipality, i.e., municipalities with fewer than 50,000 inhabitants have higher proportions of home deaths and ill-defined causes of death as shown in Table 4. These findings are probably related to better access to hospital treatment in larger cities.

Recent evidence from an ecological study demonstrated a statistically significant negative association between the Family Health Program (FHP) coverage levels and the under-5 mortality rate due to ill-defined causes in Brazil after controlling for socio-demographic characteristics (fertility rate, percentage of illiterates, and per capita income) and population size [26]. This program is a strategy of primary health care adopted in 1994 by the Brazilian Ministry of Health operating through a multiprofessional team that covers a defined geographical area of about 3,500 inhabitants with active home visits by community health workers and, when necessary, by physicians as well [26,27]. In 2008 the FHP was present in 94% of Brazilian municipalities [26]. In accordance with this high estimate, it is important to note that the majority of deceased persons in our study had the FHP coverage, so they had access to health facilities.

It is possible that the FHP has less impact on the quality of VR in low-income municipalities [26] due to the lack of qualified human resources, allowing weaknesses in identifying the cause of death, and errors in the procedures of death notification to the MIS at the municipal level [7]. Thus, administrative errors in passing the information on from a local level to the appropriate level is a major possibility, as we also found some nonrecorded death certificates in registry offices with injury as a cause of death. In addition, in our study we found that some registered ill-defined causes of death had defined causes after hospital investigation. Although the investigation of ill-defined causes of death considering hospitals, forensic institutes, and home interviews has been advocated as a simple methodology of information recovery [2], we should also point out the scarcity of systematic studies in small communities in the country. In poor-resource areas, where training of health professionals may be a challenge, we understand that all possible sources of medical/health information about the disease or death should be considered. In our investigation, this information was previously reviewed before conducting VA interviews with the family members. Despite relatively good access to health care in the region, our findings indicate a possible weak link between health care activities and the MIS, and they highlight the importance of standardizing the data collection process at the municipal level to improve the quality of the VR system.

In Brazil, several studies have been undertaken for validating the medical certification of causes of death [20]. However, very few studies have investigated the distribution of reclassified causes of death among ill-defined conditions. In our study, as indicated above, we found that a high proportion of ill-defined deaths in small municipalities were due to injuries (12.6%), and we think that this finding could well be related to the poor quality in filling out death certificates and/or problems related to the data flow at a municipal level. A previous field study in 13 heterogeneous municipalities in the states of Sergipe, Mato Grosso, and São Paulo and other research in the state of Rio de Janeiro using data from hospital admissions forms have also found injury among ill-defined conditions [2,3].

Although we cannot be certain that our results can be generalized for the whole northeast region of Minas Gerais, we understand that we are able to suggest a comparison of the VR data taking into account the estimate of the proportion of ill-defined causes reclassified as due to injury. Thus, if we apply the 12.6% adjustment factor to VR data in this region, out of the 1,393 ill-defined causes before adjusting, 175 cases should be due to injury. Then after adjusting, we should have 674 injury deaths (the pre-adjustment number of injuries was 499), and the adjusted injury fraction should be 12.5%. That is to say, before adjusting, the injury fraction on VR data was 9.2% (Table 4), compared to the 12.5% after adjusting. However, some characteristics of the losses could affect these results. Household interviews were particularly less successful for the deceased resident in rural areas and without coverage by the FHP, and we had higher proportion of males among nonparticipants in the study sample. But we believe that the losses are unlikely to significantly affect the study findings.

In general, injury is not considered in the redistribution procedures of ill-defined conditions, as more complete registration of deaths from this cause is expected [28,29]. Thus, we hope that our findings help to clarify the importance of using empirical studies to adjust VR data and derive "best" estimates of injuries in areas with data quality problems. Our results may have important implications for policy planning as injury is considered a public health concern in urban areas of high population density, and there is some evidence that violence has been spreading to the interior of the country [9].

Data from other countries also indicate that this problem is not unique to Brazil. Some studies from Oklahoma and Boston in the United States have detected inaccuracy of death certificates that may underestimate the contribution of injury to elderly mortality [30,31]. A cross-sectional study in Iran of six leading ill-defined causes of death also detected that 10% and 5% of the deaths were due to injuries for the age groups of 15-69 years old and 70 years old and over, respectively. According to the authors, a possible explanation for these findings might be that injury deaths of undetermined intent pending investigation by the Iranian Legal Medicine Organization were coded as ill-defined conditions [32]. In Brazil, the cause of death is certified by a coroner if there are suspicious circumstances surrounding the event. It is possible that in small municipalities the availability of this medical examiner is less frequent and the cause of death is declared as undetermined by a physician or even by the civil registrar, in which case the cause of death is usually not recorded.

In this study, although our eligible sample of 202 ill-defined deaths was actually based on a sampling frame for the whole year of 2007, the population source available in April 2008 when we started the field research (n = 1,124 ill-defined causes) did not include the total number of ill-defined deaths registered in 2007 in the Northeast region of Minas Gerais, due to changes in the reporting process deadline of the final data to the health department. The final numbers (n = 1,393 ill-defined deaths, all deaths = 5,409) became available from the Ministry of Health only in March 2009. It was not possible to consider in our research the ill-defined deaths included (n = 269) due to practical and logistic reasons. However, we believe that this constraint did not interfere in selection bias as the proportional cause of death distribution was almost similar in both groups (data not presented).

Although the proportions of injuries in the state of Minas Gerais, southeast region, and Brazil as a whole were relatively similar, this proportion in northeastern Minas Gerais was lower. This area has a higher proportion of ill-defined causes of death, and it seems that the fraction of injuries is inversely related to the proportion of ill-defined causes of death (as seen in Table 4), probably due to better registration of injuries on VR. On the other hand, the proportion of injuries in our study sample (4.4%) is lower when compared with the entire northeast region of Minas Gerais (9.2%). A possible explanation for this observation is the occurrence of misclassification due to higher fractions of ill-defined causes in the sampled municipalities.

This study also has some other limitations concerning the time period between death and VA interview. Information from VA interviews was collected as far back as 13 months, on average. Although possible, we consider that the effect of recall period is less important for injury causes, as misclassification has been less observed among injuries [33,34]. Information about the disease or death (health worker information and/or hospital visits) was available for almost half of the cases (n = 72) and this was used to complement VA data, thus helping the medical professional to assign the underlying cause of death. Although this proportion is similar for injury, in only four cases was it related to violence or accidents, and in one unique case the final diagnosis would not be injury if based only on the VA data. Thus, the application of standardized VA procedures has significant potential for improving knowledge on causes of death in small municipalities and should be conducted in such a setting as to complement the VR. It is also important to note that active research for additional documented information could reveal weaknesses in collecting and processing mortality data of the MIS at the municipal level.

In conclusion, the findings of this study suggest that the use of VA procedures for diagnosing causes of death among recorded ill-defined conditions might provide information on the relevance of injury as a priority health problem in small municipalities in Brazil. VA can be conducted to correct for the large proportion of ill-defined deaths in the vital registration system and estimate the additional burden of violence and accidents. Therefore, for these areas it might be more appropriate to apply the defined cause-specific fractions from VA when dealing with ill-defined deaths. Beyond a better understanding of causes of death, the use of VA in these areas can also help to check the quality of the existing VR system. Thus, VA is an important tool in the investigation of ill-defined conditions in existing VR systems with data quality problems. It could represent a feasible midterm strategy for improving mortality information when the proportion of ill-defined causes is important, such as in small Brazilian municipalities, until the MIS reaches adequate levels of quality on the definition of the underlying cause of death [7]. On the other hand, our results emphasize the need to investigate other specific populations to better understand the true causes of these deaths. Local research with VA should be brought to the attention of regional health policymakers to improve the quality of data for their planning.

## Conflicts of interest

The author declares that they have no competing interests.

## Authors' contributions

EF, DC, and MFMS participated in the conception and design of the study. EF and DC performed the analysis of the data. EF, DC, and MDCG participated in the discussion and interpretation of the results. EF wrote the first draft of the manuscript, and MDCG reviewed the manuscript. All authors read, contributed to, and approved the final manuscript.
